# X-ray diffraction analysis and *in vitro* characterization of the UAM2 protein from *Oryza sativa*

**DOI:** 10.1107/S2053230X17004587

**Published:** 2017-03-29

**Authors:** Ditte Hededam Welner, Alex Yi-Lin Tsai, Andy M. DeGiovanni, Henrik Vibe Scheller, Paul D. Adams

**Affiliations:** a DTU Bioengineering, Technical University of Denmark, Elektrovej, Building 375, 2800 Lyngby, Denmark; b Joint BioEnergy Institute, Emeryville, CA 94608, USA; c Molecular Biophysics and Integrated Bioimaging Division, Lawrence Berkeley National Laboratory, One Cyclotron Road, Berkeley, CA 94720, USA; d Biological Systems and Engineering Division, Lawrence Berkeley National Laboratory, One Cyclotron Road, Berkeley, CA 94720, USA; e Department of Plant and Microbial Biology, University of California Berkeley, Berkeley, CA 94720, USA; f Department of Bioengineering, University of California Berkeley, Berkeley, CA 94720, USA

**Keywords:** reversibly glycosylated polypeptide, limited proteolysis, UDP-arabinopyranose mutase, vector data collection, limited proteolysis

## Abstract

The role of seemingly non-enzymatic proteins in complexes interconverting UDP-arabinopyranose and UDP-arabinofuranose (UDP-arabinosemutases; UAMs) in the plant cytosol remains unknown. To shed light on their function, crystallographic and functional studies of the seemingly non-enzymatic UAM2 protein from *Oryza sativa* (OsUAM2) were undertaken. Here, X-ray diffraction data are reported, as well as analysis of the oligomeric state in the crystal and in solution. OsUAM2 crystallizes readily but forms highly radiation-sensitive crystals with limited diffraction power, requiring careful low-dose vector data acquisition. Using size-exclusion chromatography, it is shown that the protein is monomeric in solution. Finally, limited proteolysis was employed to demonstrate DTT-enhanced proteolytic digestion, indicating the existence of at least one intramolecular disulfide bridge or, alternatively, a requirement for a structural metal ion.

## Abstract 2

The UAM2 protein from *O. sativa* was cloned, expressed, purified and crystallized, and a complete data set was obtained from the radiation-sensitive crystals by low-dose vector data collection. In addition, it is shown that UAM2 is likely to exist as a monomer in solution and contains at least one intramolecular disulfide bridge or, alternatively, a structural metal ion.

## 1. Introduction   

UDP-arabinopyranose mutases (UAMs) constitute a class of enzymes that interconvert UDP-arabinopyranose and UDP-arabinofuranose. This activity was initially identified through the fractionation of rice-seedling extracts and monitoring of UDP-arabinopyranose mutase activity (Konishi *et al.*, 2007 ▸). Enzymes with UAM activity have been identified in rice, mung bean, *Arabidopsis thaliana*, green algae and wheat (Hsieh *et al.*, 2016 ▸; Konishi *et al.*, 2007 ▸; Kotani *et al.*, 2013 ▸; Rautengarten *et al.*, 2011 ▸), indicating that this activity is evolutionarily conserved within the plant kingdom. While the biological function of UAMs remained elusive until recently, studies now indicate a role in cell-wall biosynthesis; UAM knockdown transformants with decreased cell-wall arabinose content were generated in *A. thaliana* (Rautengarten *et al.*, 2011 ▸) and *UAM* gene downregulation results in decreased cell-wall arabinose in switchgrass (Willis *et al.*, 2016 ▸). Furthermore, *A. thaliana* UAMs were found to be associated with the Golgi apparatus, which is the primary site of cell-wall polysaccharide biosynthesis (Delgado *et al.*, 1998 ▸; Rautengarten *et al.*, 2011 ▸).

UAMs are members of the reversibly glycosylated polypeptide (RGP) family of proteins and autoglycosylate a conserved arginine residue using nucleotide sugars as donor substrates. Like UAMs, RGP proteins have been shown to be associated with the Golgi apparatus in pea (Dhugga *et al.*, 1997 ▸), potato (Bocca *et al.*, 1999 ▸) and cotton (Zhao & Liu, 2002 ▸). The autoglycosylation activity is thought to be coupled to the mutase activity, since mutating the conserved arginine residue abolished the catalytic activity of UAMs from *Oryza sativa* (Konishi *et al.*, 2010 ▸).

*A. thaliana*, wheat, rice and potato each contain 2–5 UAM homologs, which have been shown to exist in protein complexes *in planta* (Langeveld *et al.*, 2002 ▸; Drakakaki *et al.*, 2006 ▸; Konishi *et al.*, 2007 ▸; De Pino *et al.*, 2007 ▸; Rautengarten *et al.*, 2011 ▸). Interestingly, some of these homologs encode seemingly non-enzymatic UAMs, which share around 45% sequence identity with their catalytically active counterparts. In addition to their lack of mutase activity, these proteins also seem to be deficient in autoglycosylation activity, even though they do contain the conserved arginine that is normally the glycosylation site. The role of seemingly non-enzymatic UAM homologs in catalytically active UAM complexes is unclear and could be regulatory or related to structural scaffolding. Rice contains three UAM proteins (OsUAMs), but activity has been detected only for OsUAM1 and OsUAM3, whereas OsUAM2 seems to be deficient in UDP-arabinose mutase and autoglycosylation activities (Konishi *et al.*, 2007 ▸).

To shed light on the seemingly non-enzymatic UAMs, we have cloned, expressed and crystallized the protein OsUAM2 from *O. sativa* (rice). A sequence search of the Protein Data Bank (http://www.rcsb.org; Berman *et al.*, 2000 ▸) reveals no similarity of the OsUAM2 sequence to any solved structure, and thus structure solution is most likely to require experimental phasing.

## 2. Materials and methods   

### 2.1. Macromolecule production   

The *OsUAM2* gene was cloned into the donor vector with the pENTR Directional TOPO Cloning Kit (Thermo Fisher, catalog No. K240020), and subsequently into the expression vector with LR Clonase II (Thermo Fisher, catalog No. 11791020). The expression vector produces the target protein with an N-terminal hexahistidine tag followed by maltose-binding protein and a *Tobacco etch virus* (TEV) protease cleavage site. OsUAM2 was expressed in 0.5 l autoinduction medium (Studier, 2005 ▸), incubating at 18°C for 72 h in an Infors HT Multitron Pro with shaking at 200 rev min^−1^. The cells were resuspended in 25 ml lysis buffer (25 m*M* HEPES, 300 m*M* NaCl, 5 m*M* MgCl_2_, 1 m*M* DTT, 10 m*M* imidazole, 0.1 mg ml^−1^ hen egg-white lysozyme, 10 µg ml^−1^ DNAse I, 1 m*M* PMSF pH 8.0), incubated for 20 min at room temperature with stirring and lysed by two passes through a French press. The cleared lysate was loaded onto a 5 ml HisTrap FF nickel affinity column (GE Life Sciences, catalog No. 17-5255-01) and eluted with 20 column volumes of elution buffer (25 m*M* HEPES, 300 m*M* NaCl, 250 m*M* imidazole pH 8.0). The pooled elution fractions were then subjected to proteolytic cleavage with hexahistidine-tagged TEV protease for 2 h at room temperature. TEV protease and uncleaved fusion protein were removed by another pass through the HisTrap column, and the salt in the flowthrough was then adjusted to 100 m*M* NaCl. This sample was fractionated on a HiTrap Q HP anion-exchange column (GE Life Sciences, catalog No. 29-0513-25), eluting with a linear gradient from 100 to 1000 m*M* NaCl over 20 column volumes. The fractions were analyzed by SDS–PAGE and pooled accordingly. The pooled preparation was then analyzed by SDS–PAGE and estimated to be at least 90% pure by visual inspection of the Coomassie-stained gel. The *A*_260_/*A*_280_ absorbance ratio of the preparation was measured to be 1.9, indicating no problem with DNA contamination. The absorbance of the preparation at 280 nm was 26 AU, which together with a calculated theor­etical extinction coefficient of OsUAM2 of 1.3 ml mg^−1^ cm^−1^ gives a protein concentration of 20 mg ml^−1^. Macromolecule-production information is summarized in Table 1 ▸.

### 2.2. Crystallization   

**Figure 1:**
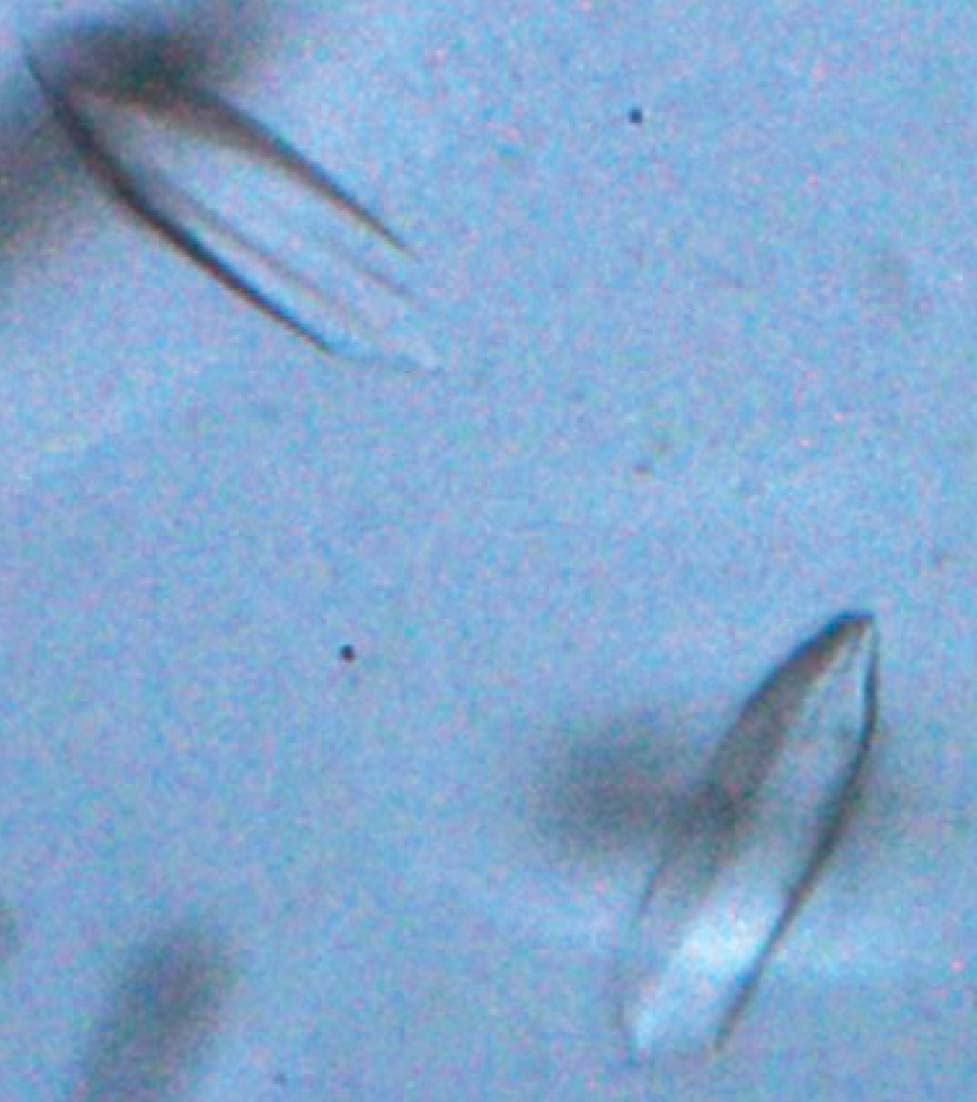
Crystals of OsUAM2 grown as described. The largest dimension is approximately 100 µm.

Six commercially available screens were employed: Crystal Screen (Hampton Research, catalog No. HR2-110), Natrix (Hampton Research, catalog No. HR2-116), Index (Hampton Research, catalog No. HR2-144), PEG/Ion (Hampton Research, catalog No. HR2-126), PEGRx 1 (Hampton Research, catalog No. HR2-082) and MCSG1 (Anatrace, catalog No. MCSG-1T). The screens were set up with a Phoenix liquid-handling system (Art Robbins Instruments). Crystals of uniform morphology formed within 2 d in ∼5% of the screened conditions (Fig. 1 ▸); extensive crystal-optimization efforts did not improve the crystal quality compared with the initial crystals. Crystallization information is summarized in Table 2 ▸.

### 2.3. Data collection and processing   

Single crystals were transferred with a mounted CryoLoop (Hampton Research) into a 10 µl drop of reservoir solution containing 22%(*v*/*v*) glycerol for cryoprotection and were then flash-cooled in liquid nitrogen. Initial diffraction tests revealed these crystals to be very radiation-sensitive, with severe loss of diffraction power over the course of data collection. In addition, high-resolution diffractions were also lost with decreased dose, *i.e* when attenuating the beam or decreasing the exposure time. The chosen data-collection parameters (no attenuation and 1 s exposure per frame) represent the best compromise identified between low dose and resolution limit. With these settings, diffraction is lost after about 30°. To achieve complete data, 25° of data were collected at each of three vector points. The data from the third vector point showed several severe pathologies, including multiple lattices and decreased diffraction power, and consequently these data were discarded. Data from the two remaining vector points were processed separately with *iMosflm* (Battye *et al.*, 2011 ▸) and then combined with *CCP*4 (Winn *et al.*, 2011 ▸). Data quality was assessed with *phenix.xtriage* (Zwart *et al.*, 2005 ▸; Adams *et al.*, 2010 ▸). The space group was assigned with *POINTLESS* (Evans, 2011 ▸). Matthews parameters (Matthews, 1968 ▸) were calculated using *MATTHEWS_COEF* as implemented in *CCP*4 (Kantardjieff & Rupp, 2003 ▸). Data-collection and processing statistics are summarized in Table 3 ▸.

### 2.4. Analytical size-exclusion chromatography   

Purified OsUAM2 was centrifuged and injected onto a Superdex 200 10/300 GL size-exclusion column (GE Life Sciences, catalog No. 17517501) using an ÄKTAexplorer 100 FPLC system (GE Life Sciences, catalog No. 18111241) and a flow rate of 0.5 ml min^−1^. The column was pre-equilibrated in running buffer (25 m*M* HEPES, 300 m*M* NaCl, 5 m*M* MgCl_2_ pH 8.0 with or without 1 m*M* DTT). Protein standards (Bio-Rad, catalog No. 151-1901) were run in the same buffers and with the same flow rate to ensure accurate results.

### 2.5. Limited proteolysis   

OsUAM2 at 1 mg ml^−1^ in proteolysis buffer (25 m*M* HEPES, 300 m*M* NaCl, 5 m*M* MgCl_2_, 6 µ*M* CaCl_2_ pH 8.0 with or without 1 m*M* DTT) was equilibrated on ice for 30 min. Subtilisin (Sigma–Aldrich, catalog No. P-5380) was diluted from a 3.5 mg ml^−1^ stock in 100 m*M* Tris–HCl pH 8.0 to 100 and 10 ng ml^−1^ in the proteolysis buffer. Subtilisin and OsUAM2 were mixed in various volume ratios to achieve the desired mass ratios (0–6% subtilisin:OsUAM2) and incubated on ice for 30 min. At this point, SDS sample buffer (5× SDS–PAGE sample buffer: 125 m*M* Tris–HCl pH 6.8, 2% SDS, 20% glycerol, 0.4% bromophenol blue) and 100 m*M* DTT were added to the samples, which were then incubated at 95°C for 10 min. The samples were then cooled, centrifuged and analysed by SDS–PAGE using 4–20% Mini-PROTEAN TGX precast gels (Bio-Rad, catalog No. 4561096), which were subsequently stained with InstantBlue colloidal Coomassie dye (Expedeon, catalog No. ISB1L).

## 3. Results and discussion   

**Figure 2:**
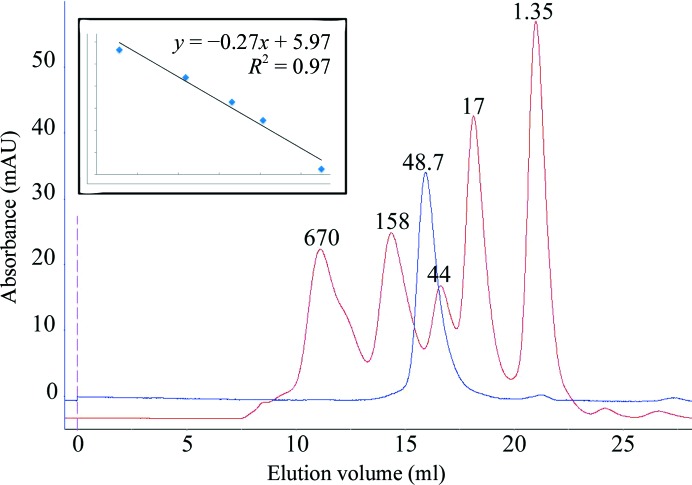
Analytical size-exclusion chromatography. Chromatograms of OsUAM2 (blue) and molecular-weight standards (red). The peaks are annotated with the known (molecular-weight standards: thyroglobulin, 679 kDa; γ-­globulin, 158 kDa; ovalbumin, 44 kDa; myoglobin, 17 kDa; vitamin B_12_, 1.35 kDa) or calculated (OsUAM2, 48.7 kDa) molecular weights. The insert shows the standard curve used for column calibration.

We have cloned, expressed, purified and crystallized the OsUAM2 protein from *O. sativa*. This protein crystallizes readily in a variety of conditions (Fig. 1 ▸). However, the crystals are of limited quality, diffracting to a maximum of 3.6 Å resolution, quickly deteriorating with radiation dose and frequently displaying a mixed lattice diffraction pattern. Extensive optimization efforts, including seeding, limited proteolysis and online dehydration/rehydration experiments, have largely failed. OsUAM2 is predicted to be folded, adopting both α-helical and β-sheet structure elements and containing no significant regions of disorder or trans­membrane domains that could guide construct optimization. However, employing a vector data-collection strategy, we have succeeded in obtaining a 98.9% complete data set, leveraging the high symmetry of the tetragonal space group. *Phenix.xtriage* (Zwart *et al.*, 2005 ▸; Adams *et al.*, 2010 ▸) analysis indicates the presence of weak ice rings and no twinning or translational noncrystallographic symmetry. Given the limited number of data points, space-group assignment was carefully considered. While the Laue group was unambiguously identified, the statistics cannot clearly discriminate between the possible space groups. The top solution from *POINTLESS* (Evans, 2011 ▸) analysis is *P*4_2_2_1_2, which gives an average signal-to-noise ratio for the systematic absences of −2.2 and 0.06 for the fourfold axis and twofold axis, respectively. This seems to be valid for the twofold axis, where 16 out of 18 expected absences have a signal-to-noise ratio of below 3. However, for the fourfold axis the number of relevant data points is so low that, even though there are no absence violations, unambiguous space-group determination will have to await phasing. Matthews analysis shows that the unit-cell parameters and space-group symmetry together with the OsUAM2 amino-acid sequence are compatible with the presence of one to three molecules per asymmetric unit. A self-rotation analysis shows a weak 4.6σ peak corresponding to a twofold rotation at θ = 90° and φ = 120°. This, together with the limited diffraction power of the crystals, is suggestive of the presence of two molecules in the asymmetric unit, with a solvent content of 63% and a Matthews coefficient of 3.29 Å^3^ Da^−1^. To investigate whether this could be indicative of a functional dimer, the oligomerization state of OsUAM2 in solution was probed by size-exclusion chromatography. The chromatogram indicates that OsUAM2 is monomeric in solution under the given conditions (Fig. 2 ▸), eluting as a single, symmetric peak corresponding to a molecular weight of 48.7 kDa. This is within reasonable agreement with the calculated molecular weight of 40.1 kDa.

**Figure 3:**
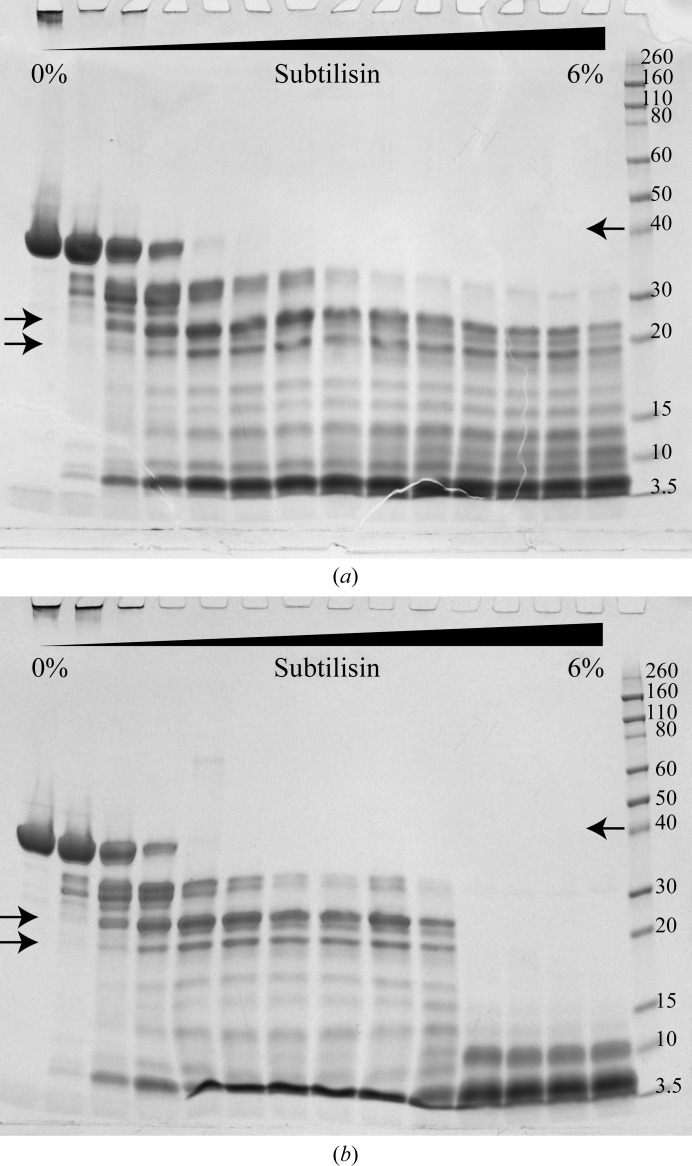
Limited proteolysis of OsUAM2 in the absence (*a*) and presence (*b*) of DTT with varying mass ratios of subtilisin to OsUAM2. The arrows on the right indicate the migration length of full-length OsUAM2, while the arrows on the left indicate the two dominant digestion products that were shown to represent overlapping digestion products by N-terminal sequencing.

To further probe the conformational features of OsUAM2, and possibly identify stable subdomain(s) that could potentially yield higher quality crystals, we subjected purified OsUAM2 to limited proteolytic digestion. Intermediate digestion preserving a number of species up to 30 kDa was readily achieved. Two predominant bands with apparent molecular weights of approximately 25 and 18 kDa were speculated to represent two subdomains of the full-length protein (Fig. 3 ▸ *a*). These were analysed by N-terminal sequencing, which showed that both bands originated from overlapping N-terminal fragments of different lengths and thus did not represent distinct, stable subdomains (data not shown).

Varying the salt concentration between 100 and 500 m*M* did not alter the proteolytic digestion pattern. In contrast, adding DTT to the digestion mixture enhanced digestion significantly (Fig. 3 ▸ *b*). DTT-mediated removal of a structural metal ion could possibly explain this effect, since rice UAMs have been shown to require Mn^2+^ for activity (Konishi *et al.*, 2007 ▸). However, many enzymes with nucleotide substrates, including glycosyltransferases such as the UAMs, require Mg^2+^ or Mn^2+^ for activity, and these ions have been shown to interact with the nucleotide diphosphate group in the active site, with no role in the structural integrity of the protein itself implied (Lairson *et al.*, 2008 ▸). This, together with the facts that we have an excess of Mg^2+^ in the proteolysis buffer (5 m*M* MgCl_2_ to 1 m*M* DTT) and that OsUAM2 remains soluble in the presence of DTT during size-exclusion chromatography (see below), makes the removal of a structural metal ion a less likely explanation. Rather, we believe the observed DTT-mediated proteolytic sensitivity can be attributed to the reducing activity of DTT. Subtilisin (the protease) and OsUAM2 contain zero and seven cysteines, respectively, thus indicating that the observed effect reflects the reduction of one or more protective disulfide bridges in OsUAM2. While disulfide bridges in cytosolic proteins might be rare, the Protein Data Bank currently contains the structures of seven unique cytosolic *A. thaliana* proteins with at least one disulfide brige (http://www.rcsb.org; Berman *et al.*, 2000 ▸).

To investigate whether the disulfide bridge(s) is/are intermolecular or intramolecular, we revisited size-exclusion chromatography. The initial analysis had indicated that OsUAM2 is monomeric in solution, but OsUAM2 did elute a little faster than expected, corresponding to a slightly higher molecular weight than the monomer (48.7 kDa compared with 40.1 kDa). However, on adding DTT to the protein preparation and the size-exclusion running buffer we observed no shift in elution volume (data not shown). This led us to conclude that the seven cysteines in the OsUAM2 amino-acid sequence form at least one intramolecular disulfide bridge that partially shields the protein from proteolytic digestion. Whether this disulfide bridge is native or a result of the purification procedure cannot be determined.

## Figures and Tables

**Table 1:** Macromolecule-production information

Source organism	*O. sativa*
DNA source	Tiller cDNA (prepared in-house)
Forward primer	CACCATGTCTTTGGAAATTCAGGACAGT
Reverse primer	AGCGCTTTGAGCTCCGTGAGATTT
Cloning vector	pENTR/SD/D-TOPO (Thermo Fisher, catalog No. K240020)
Expression vector	pVP16 (Aceti *et al.*, 2015 ▸)
Expression host	*E. coli* BL21 (DE3)
Complete amino-acid sequence of the construct produced†	MGHHHHHHHHSKIEEGKLVIWINGDKGYNGLAEVGKKFEKDTGIKVTVEHPDKLEEKFPQVAATGDGPDIIFWAHDRFGGYAQSGLLAEITPDKAFQDKLYPFTWDAVRYNGKLIAYPIAVEALSLIYNKDLLPNPPKTWEEIPALDKELKAKGKSALMFNLQEPYFTWPLIAADGGYAFKYENGKYDIKDVGVDNAGAKAGLTFLVDLIKNKHMNADTDYSIAEAAFNKGETAMTINGPWAWSNIDTSKVNYGVTVLPTFKGQPSKPFVGVLSAGINAASPNKELAKEFLENYLLTDEGLEAVNKDKPLGAVALKSYEEELAKDPRIAATMENAQKGEIMPNIPQMSAFWYAVRTAVINAASGRQTVDEALKDAQTNSSSNNNNNNNNNNLGIDENLYLENLYFQGTSLYKKAGFKMSLEIQDSEVDIVIAALQPNLTTFFEAWRPFFSRFHIIVVKDPDMAEELQIPTGFDLKVYTKSDMGVLGATSIDFSGHSCRYFGYLVSRKKYVISIDDNCLPAKDNGGLTVDAVAQHMSNLKTPATPFFFNTLYDPFRKGADFVRGYPFSLREGVECMLSCGLWLHNADYDPMTHVVKRNQRNTTYVDAVMTVPLGAMMPVSGINVAFNREVLGPVMFPALRLRKEGKHRWDTLEDVWNGLCAKVVCDRLRYGVKTGLPYVMRSDAEAGKALESLKEWEGVKVMDVVLPFFESLKLSSTSVTVEDCVKELTSIVKEKLGPQNAIFAKAADAMEEWTKLWKSHGAQSA

† The OsUAM2-containing cleavage product is underlined.

**Table 2:** Crystallization

Method	Sitting-drop vapour diffusion
Plate type	3-well Intelli-Plate (Art Robbins Instruments)
Temperature (K)	294
Protein concentration (mg ml^−1^)	5
Buffer composition of protein solution	25 m*M* HEPES pH 8.0, 100 m*M* NaCl, 5 m*M* MgCl_2_
Composition of reservoir solution	100 m*M* HEPES pH 7.5, 25%(*v*/*v*) PEG3350, 200 m*M* MgCl_2_·6H_2_O
Volume and ratio of drop	0.2 µl protein + 0.2 µl reservoir
Volume of reservoir (µl)	100

**Table 3:** Data collection and processing Values in parentheses are for the outer shell.

Diffraction source	Beamline 5.0.1, Advanced Light Source
Wavelength (Å)	0.97741
Temperature (K)	100
Detector	3 × 3 CCD array, ADSC Q315r
Crystal-to-detector distance (mm)	449
Rotation range per image (°)	1
Total rotation range (°)	50
Exposure time per image (s)	1
Space group	*P*4_2_2_1_2
Unit-cell parameters (Å, °)	*a* = *b* = 115.4, *c* = 157.9, α = β = γ = 90
Mosaicity (°)	0.6
Resolution range (Å)	65.2–3.6 (3.8–3.6)
Total No. of reflections	23422 (2272)
No. of unique reflections	12771 (1256)
Completeness (%)	98.9 (99.6)
Multiplicity	1.8 (1.8)
〈*I*/σ(*I*)〉	5.3 (1.7†)
*R*_r.i.m._	0.197 (0.831)
Overall *B* factor from Wilson plot (Å^2^)	73.4

† The data were cut according to CC_1/2_ > 0.3 in the outer shell. At the chosen cutoff (3.6 Å), CC_1/2_ = 0.34. *I*/σ(*I*) falls below 2 at 3.7 Å resolution.
